# Parenteral and oral antibiotic duration for treatment of pediatric osteomyelitis: a systematic review protocol

**DOI:** 10.1186/2046-4053-2-92

**Published:** 2013-10-07

**Authors:** Chelsey Grimbly, Jeff Odenbach, Ben Vandermeer, Sarah Forgie, Sarah Curtis

**Affiliations:** 1 Department of Pediatrics, University of Alberta, Edmonton Clinic Health Academy, 11405-87 Avenue, Edmonton, AB T6T 1C9, Canada; 2 Women & Children’s Health Research Institute, University of Alberta, 4–081 Edmonton Clinic Health Academy (ECHA), 11405-87 Avenue, Edmonton, AB T6G 1C9, Canada; 3 Alberta Research Centre for Health Evidence Room 4-496A, 4th Floor, Edmonton Clinic Health Academy, 11405-87 Avenue, Edmonton, AB T6G 1C9, Canada

## Abstract

**Background:**

Pediatric osteomyelitis is a bacterial infection of bones requiring prolonged antibiotic treatment using parenteral followed by enteral agents. Major complications of pediatric osteomyelitis include transition to chronic osteomyelitis, formation of subperiosteal abscesses, extension of infection into the joint, and permanent bony deformity or limb shortening. Historically, osteomyelitis has been treated with long durations of antibiotics to avoid these complications. However, with improvements in management and antibiotic treatment, standard of care is moving towards short durations of intravenous antibiotics prior to enteral antibiotics.

**Methods/Design:**

The authors will perform a systematic review based on PRISMA guidelines in order to evaluate the literature, looking for evidence to support the optimal duration of parenteral and enteral therapy. The main goals are to see if literature supports shorter durations of either parenteral antibiotics and/or enteral antibiotics.

Multiple databases will be investigated using a thorough search strategy. Databases include Medline, Cochrane, EMBASE, SCOPUS, Dissertation Abstracts, CINAHL, Web of Science, African Index Medicus and LILACS. Search stream will include medical subject heading for pediatric patients with osteomyelitis and antibiotic therapy. We will search for published or unpublished randomized and quasi-randomized controlled trials.

Two authors will independently select articles, extract data and assess risk of bias by standard Cochrane methodologies. We will analyze comparisons between dichotomous outcomes using risk ratios and continuous outcomes using mean differences. 95% confidence intervals will be computed.

**Discussion:**

One of the major dilemmas of management of this disease is the duration of parenteral therapy. Long parenteral therapy has increased risk of serious complications and the necessity for long therapy has been called into question. Our study aims to review the currently available evidence from randomized trials regarding duration of both parenteral and oral therapy for pediatric acute osteomyelitis.

**Trial registration:**

CRD42013002320

## Background

### Description of the condition

Osteomyelitis is inflammation of the bone, usually caused by an infectious agent. It can originate from trauma or surgery or it can originate from hematogenous seeding. Acute osteomyelitis presents with less than 2 weeks of symptoms whereas subacute infections have symptoms that persist for two weeks or more and chronic infections last months or longer.

The incidence is 1:5,000 to 3:100,000 in the pediatric population, and it is most common in the neonatal and pre-school periods [1-3]. In the pre-antibiotic era, children had a mortality rate of over 23% [4], a rate that dropped to 3.5% with the introduction of antibiotics. Today, in the developed world, clinicians see minimal morbidity and almost no mortality with treatment. However, the developing world still sees a high morbidity of osteomyelitis with challenges of inadequately treated osteomyelitis, the development of chronic infections and bony sequestration, and associated deformities and pathological fractures [5,6].

In children, the most common sites of infection are the femur (27%), tibia (24%), and pelvis (15%) [7]. Diagnosis is largely clinical with classical physical features of pain, tenderness, swelling over a bony site and fever. A variety of laboratory tests may be abnormal with raised erythrocyte sedimentation rate (ESR) (91%), raised C-reactive protein (CRP) (80%) and raised white blood cell count (35%) being the most commonly examined. Radiological tests may also be helpful to support the diagnosis with the use of magnetic resonance imaging (MRI), ultrasound, bone scan and x-rays as commonly used modalities. Blood cultures are often done and occasionally bony aspirates or biopsies at suspected site of infection are done [8].

*Staphylococcus aureus* is the causative agent in 80% of cases although there are less common bacterial agents including *Haemophilus influenzae*, *Streptococcus pyogenes*, *Escherchia coli* among others [3,9]. Neonatal osteomyelitis is a separate entity with Group B streptococcus being the main causative agent, patients are 40% more likely to have multiple infected sites, and they have more subtle presentations. Infections are also more commonly secondary to line sepsis [10]. Complications of osteomyelitis include the development of a chronic bony infection, subperiosteal abscess formation, venous thrombosis [11] and secondary septic arthritis. Septic arthritis is a surgical emergency requiring urgent drainage and long term complications can include joint stiffness, joint dislocation, and limb shortening.

### Description of the intervention

Osteomyelitis requires prompt antimicrobial treatment starting with intravenous antibiotics before transitioning to enteral antibiotics to avoid complications that include periosteal abscess and the development of chronic osteomyelitis with associated bony deformities [12,13]. However, there is an absence of consensus regarding antibiotic duration, with most sources supporting conservative prolonged management. Review articles and notable pediatric textbooks recommend an average of 1 to 2 weeks of parenteral antibiotics until the C-reactive protein normalizes, and a full 3 to 6 weeks of total antibiotics until the erythrocyte sedimentation rate normalizes [13-15]. This may require central venous catheter insertion, a prolonged hospitalization, and extensive cost to the family and healthcare system. Other centers initiate several days of peripheral intravenous (IV) antibiotics prior to inserting a central venous catheter and then provide 4 to 6 weeks of home IV therapy [16].

The risk of complications associated with a central line include line malfunction or displacement, catheter associated bloodstream infection, fever with negative blood culture results, and local skin infection [16]. As children with osteomyelitis tend to be younger than 5 years [2,14], anesthesia and the associated risks are necessary for the insertion of the central line. In centers that provide home intravenous therapy, the cost of IV antibiotics is immensely higher than that of oral [12,13].

A systematic review published in 2002 by Le Saux showed that a 7-day course of parenteral antibiotic was as effective as longer durations, with the same rate of recurrence at 6 months. However, Le Saux’s study included observational studies primarily from the 1970s and 1980s and only one study was randomized [17]. Dartnell *et al*. published a comprehensive scoping review of the diagnosis and management of pediatric osteomyelitis in 2012 [18]. That provided a general overview of the epidemiology of osteomyelitis but did not specifically address questions around the treatment of osteomyelitis in a systematic way. Our study will ask very specific questions about treatment. We will include both English and non-English studies and an expanded database search. The proposed study will involve a current systematic review of prospective randomized control studies to evaluate if long-term parenteral therapy is required or if short-term parenteral therapy prior to transition to enteral antibiotics provides safe and sufficient treatment of osteomyelitis.

### Why it is important to do this review

One of the major dilemmas of management of this disease is the duration of parenteral therapy. Long parenteral therapy has increased risk of serious complications and the necessity for long therapy has been called into question. Le Saux’s review, over 10 years ago, suggested that shorter therapies could be efficacious and safe but emphasized the need for higher quality evidence. We hypothesize that new evidence has been published over this time period. Our study aims to now review the currently available evidence from randomized trials regarding duration of both parenteral and oral therapy for pediatric acute osteomyelitis.

## Methods/Design

The PRISMA statement will be used as a template for the development and execution of this review. The systematic review protocol will be registered with the PROSPERO database before initiation. A standardized methodological approach to this systematic review will be based on guidance from the *Cochrane Handbook for Systematic Reviews of Interventions* Version 5.1.0.

### Types of studies

Studies will be limited to randomized controlled trials and quasi-randomized controlled trials that look at an intervention of intravenous antibiotic duration of less than 7 days, compared to previous standard of greater than 7 days. Studies need to describe the antimicrobial including route and duration as well as clinical outcomes with follow-up for at least 3 months. Exclusion criteria include retrospective studies and prospective non-randomized studies including case series.

### Types of participants

Pediatric patients younger than 18 years of age who have been identified with osteomyelitis will be included in this study.

### Disease definition

Acute osteomyelitis is a clinical diagnosis based on symptoms of fever, pain, and reduced weight bearing and swelling over the affected bone. The use of laboratory tests including positive blood culture, ESR and CRP, as well as diagnostic imaging such as MRI, computed tomography (CT) scan, nuclear magnetic imaging, ultrasound, or X-ray aid in supporting the diagnosis [17-19]. For this systematic review, as defined by the authors of the primary studies, we expect the definition of acute osteomyelitis to include: A) clinical signs of osteomyelitis and positive blood cultures, OR B) clinical signs and radiological support, OR C) clinical signs and positive bone culture.

### Types of interventions

We will include comparative randomized trials of antibiotics administered either parenterally or orally, or both.

### Types of outcome measures

Primary Outcome:

1. Resolution of osteomyelitis infection as determined by resolution of symptoms including pain, tenderness, swelling and gait abnormalities by end of total therapy. All outcome data will be grouped at additional discrete time periods such as 1 month, 3 months, 6 months, 1 year, and annually, if available.

Secondary outcomes:

1. Time to resolution of infection as defined above

2. Total length of antibiotic therapy

3. Residual pain, fever, swelling and other clinical features

4. Residual disabilities

5. Residual morbidities: includes limp, deformities, and neurological deficits

6. Adverse events defined as mild, moderate or severe

7. Residual radiographic changes

8. Re-hospitalization for infection

9. Re-initiation of therapy

10. Resolution of increased inflammatory marker (WBC, ESR, CRP)

11. Surgical intervention

### Literature search strategies

The systematic review will consist of a literature search of the databases Medline, EMBASE, SCOPUS, Cochrane CENTRAL Database of Systematic Reviews, CINAHL, Web of Science, Global Health, African Index Medicus and LILACS. Relevant articles will also be hand-searched for related publications. There will be no restrictions on language; any relevant non-English, non-French papers will be appropriately translated. There will be no restriction on publication period or publication status. Gray literature will be searched through Dissertation Abstracts and the library search will be repeated prior to publication to look for new citations. The major clinical trials registries will be searched and the principle investigators for any relevant registered RCT will be contacted to seek sharing of any new or unpublished trial data that may be relevant. The bibliography of all retrieved literature will be reviewed for additional reports of trials.

### Library search strategy

1. randomized controlled trial.pt.

2. clinical trial.pt.

3. randomi?ed.ti,ab.

4. placebo.ti,ab.

5. dt.fs.

6. randomly.ti,ab

7. trial.ti,ab.

8. groups.ti,ab.

9. or/1-8

10. animals/

11. humans/

12. 10 not (10 and 11)

13. 9 not 12

14. exp Infant/

15. exp Child/

16. exp Adolescent/

17. exp Pediatrics/

18. exp Young Adult/

19. exp Infant, Newborn/

20. exp Infant, Premature

21. exp Child, Preschool/

22. pubescent.mp. or exp Puberty/

23. kindergarten.mp.

24. minor*.mp.

25. prepubescent.mp.

26. (infant* or child* or teen* or adolescen* or neonat* or youth or young or p?ediatric*).mp.

27. (young adj2 adult*).mp.

28. school age.mp.

29. (student* adj2 (college* or universit*)).mp.

30. high school.mp

31. middle school.mp.

32. elementary school.mp.

33. freshman.mp.

34. sophomore.mp.

35. exp Students/

36. (child* or adolescent* or infant* or pediatric* or paediatric*).jn,jw.

37. 14 or 15 or 16 or 17 or 18 or 19 or 20 or 21 or 22 or 23 or 24 or 25 or 26 or 27 or 28 or 29 or 30 or 31 or 32 or 33 or 34 or 35 or 36

38. exp Osteomyelitis/

39. exp Bone Diseases/

40. exp Bone Diseases, Infectious/

41. exp Infection/

42. 39 and 41

43. exp Staphylococcal Infections/and (bone* or osteo*).mp. [mp = title, abstract, original title, name of substance word, subject heading word, protocol supplementary concept, rare disease supplementary concept, unique identifier]

44. exp Streptococcal Infections/and (bone* or osteo*).mp. [mp = title, abstract, original title, name of substance word, subject heading word, protocol supplementary concept, rare disease supplementary concept, unique identifier]

45. 38 or 40 or 42 or 43 or 44 or ′9′.mp. or ′10′.mp. [mp = title, abstract, original title, name of substance word, subject heading word, protocol supplementary concept, rare disease supplementary concept, unique identifier]

46. osteomyelitis.mp.

47. (bone adj3 infect*).mp.

48. exp Anti-Bacterial Agents/

49. exp Penicillins/

50. exp Cephalosporins/

51. exp Macrolides/

52. exp Quinolones/

53. exp Aminoglycosides/

54. exp Rifampin/

55. exp Glycopeptides/

56. exp Carbapenems/

57. exp tetracyclines/

58. exp Lincosamides/

59. exp Clavulanic Acids/

60. (Anti-Bacterial Agent* or antibiotic* or Penicillin* or Cephalosporin* or Macrolid* or Quinolon* or Aminoglycosid* or Rifampin* or Carbapenem* or Glycopeptid* or Tetracyclin* or Lincosamid* or Clavulinic Acid*).mp.

61. antisyphilitic drugs.mp. or exp Antitreponemal Agents/

62. hetacillin.mp.

63. cephalospor*.mp.

64. exp Lincomycin/or lincomycin.mp.

65. clavulanic acid.mp. or exp Clavulanic Acid/

66. beta lactamases.mp. or exp beta-Lactamases/

67. penicillinases.mp. or exp Penicillinase/

68. or/48-67

69. 45 and 68

70. 13 and 69

71. limit 70 to “all child (0 to 18 years)”

72. 37 and 70

73. 71 or 72

74. 45 and 73

75. 13 and 45 and 68

76. 38 or 40 or 42 or 43 or 44 or 46 or 47

77. 13 and 37 and 68 and 76

78. limit 77 to randomized controlled trial

79. limit 77 to case reports

80. 77 not 79

81. 77 not 80

### Study selection process

The literature search results will be uploaded to a reference manager EndNote X5 (Thomson Reuters, New York, NY, United States). Study selection will be done in two stages. Initially, abstracts and titles will be screened independently by two reviewers (CG and JO) against inclusion and exclusion criteria (Table 1). Both reviewers will then independently review potentially relevant papers in their entirety, deciding on inclusion using a detailed eligibility form. All reviewed and excluded articles will be recorded on an Excel spreadsheet, with reasons being noted for exclusions. If there is any discordance between the authors, the review authors will resolve any disagreements by consensus or through discussion with a third reviewer (SC), who will evaluate and decide on the contended study.

**Table 1:** Inclusion and exclusion criteria

**Inclusion**	**Exclusion**
Randomized Controlled trials	Non- randomized trials
Quasi Randomized Trials	Cohort, case-series or retrospective studies
All languages	Review articles
Acute osteomyelitis	Patients >18 years
Children 0 to 18 years	Chronic or sub-acute osteomyelitis, tuberculosis, sickle cell disease, malignancy, human immunodeficiency virus, chemotherapy
Published or unpublished	Osteomyelitis data cannot be separated from other musculoskeletal/infectious diagnoses
Antibiotic administration with follow-up for 3 months	Intervention other than antibiotic
Identify the anti-microbial and its route	Route of antimicrobial unclear
Identify duration of therapy	Duration of parenteral or enteral therapy unclear
Outcome after 3 months follow-up stated or inferred as clinical cure, failure or relapse	Follow-up not documented

### Data collection

Both authors (CG and JO) will use a standardized customized data extraction form based on and adapted from the forms supplied by the Cochrane Bone, Joint and Muscle Trauma Group. Initially the form will be piloted to ensure that data collection is consistent between both reviewers. Data to be extracted includes signs and symptoms of osteomyelitis, blood culture results, radiological support, duration of intravenous antibiotics (7 days) and qualitative total duration, duration of follow-up, whether patients were followed for 6 months after presentation, how many patients were lost to follow-up, whether central venous lines were inserted, and method of randomization.

### Risk of bias assessment

Each reviewer (CG and JO) will independently assess the risk of bias in each primary trial using the Cochrane Risk of Bias tool as described in the Cochrane Handbook for Systematic Reviews of Interventions 5.1. Each parameter will be judged as either low risk of bias, high risk of bias, or unclear risk of bias. When possible, authors will be contacted to clarify unclear criteria. Assessment of bias will be compared between authors and any discrepancies will be resolved through discussion and consensus, or through a decision by the third reviewer (SC). During data synthesis, data will be pooled together and evaluated as a whole. The data will then be re-evaluated using a sensitivity analysis to see the impact of studies with high risk of bias.

Domains of importance will include:

• Random sequence generation

• Allocation concealment

• Blinding (participants, assessors, care providers)

• Incomplete outcome data

• Selective outcome reporting

• Other sources of bias

### Data analysis

A statistician (BV) from the Alberta Research Centre for Health Evidence (ARCHE) and who is experienced with systematic review methodology has been consulted for the project. For each study, we will analyze comparisons between dichotomous outcomes using risk ratios (RR) and between continuous outcomes using mean differences. We will compute the 95% confidence intervals (CI) for all estimates. Where populations, interventions and outcomes are sufficiently clinically homogenous, results will be statistically pooled using a DerSimonian-Laird random effects model; fixed effects will be considered in a sensitivity analysis. This will be done using the Mantel-Haenszel method for RR and the inverse variance method for pooling mean differences. In cases where the outcomes are measured in different scales, a standardized mean difference (SMD) will be used to pool results, rather than a weighted mean difference (WMD). Review Manager version 5.0.22 (The Cochrane Collaboration, Copenhagen, Denmark) and Stata version 7.0 (Stata Corporation, College Station, TX, USA) will be used for all these analyses. In the event that studies cannot be pooled, evidence tables will be produced and a narrative summary of the results will be presented.

### Dealing with missing data

Authors of primary studies will be contacted to clarify any unclear or missing data.

### Assessment of heterogeneity

Heterogeneity among studies will be measured using the I^2^ statistic, which describes the percentage of total variation across trials due to heterogeneity rather than sampling error. In cases of substantial heterogeneity, subgroup and meta-regression analyses will be performed if the number of studies is sufficient to warrant these analyses. Heterogeneity will be considered to be statistically significant if I^2^ is over 25%.

### Publication bias

Where possible, we will also analyze publication bias both visually using the funnel plot and quantitatively using Egger’s regression test.

### Subgroup analysis

Subgroup analysis will be conducted for the primary outcome to assess how the effect of treatment may vary in clinically relevant subgroups. We anticipate clinical heterogeneity and thus the following a-priori clinically relevant subgroups have been identified for consideration:

Different age groups (0 to 11 months, 1 to 5 years, 6 to 12 years and 13 to 18 years old)

Microbiological cause (methicillin-resistant *Staphylococcus aureus*, Syphilis, *Gonococcal osteomyelitis*, *Borrelia burgdorferi*)

Surgical interventions

Type of antibiotic

Immunization status

Publication date

Patient origin

### Sensitivity analysis

Sensitivity analyses will be conducted to assess the robustness of the findings across study quality (low risk of bias versus high risk or unclear risk), parallel versus crossover trial designs, random effects versus fixed effects analyses, and randomized controlled trials versus quasi-randomized trials.

## Discussion

This systematic review will update the literature on best practice in pediatric osteomyelitis. A similar study was conducted in 2002 and found that shorter courses of parenteral therapy had similar cure rates to prolonged courses but the results were limited to cohort studies. We will conduct a comprehensive search reviewing an estimated 3,400 studies, compared to 284 reviewed by Le Saux *et al*. and, we hope that relevant randomized controlled trials will have been published in the past decade.

This review will allow statistical analysis of outcomes to prove whether short- or long-term parenteral antibiotics are better for clinical outcomes. If short-term parenteral antibiotics are superior, this will provide evidence to minimize hospital stay and avoid the insertion of central venous catheters. If long-term parenteral antibiotics are superior, this will provide justification to families for the strain of prolonged health care systems involvement for therapy. We hope to also find similar data regarding the optimal duration of enteral therapies.

## Abbreviations

ARCHE: Alberta Research Centre for Health Evidence; CI: Confidence interval; CRP: C-reactive protein; CT: Computed tomography; ESR: Erythrocyte sedimentation rate; IV: Intravenous; MRI: Magnetic resonance imaging; RR: Risk ratio; SMD: Standardized mean difference; WMD: Weighted mean difference.

## Competing interests

The authors declare that they have no competing interests.

## Authors’ contributions

CG is the principle investigator and project lead. She generated the idea and concept, will complete the literature search, study selection, data collection, and risk of bias assessment. JO is the second investigator. He will complete the study selection, data collection and risk of bias assessment independently of CG. SC is the project supervisor who will provide experience in evidence based medicine methodology. She contributed to the protocol and manuscript and will be a third reviewer for article inclusion. SF is an expert in Pediatric Infectious Disease who will provide expert opinion and aid in reviewing the protocol and manuscript. BC is a statistician who will lead the statistical analysis plan, assist in statistical analysis, and contribute to the protocol and manuscript. All authors read and approved the final manuscript.
